# Genome Sequence Analyses of *Pseudomonas savastanoi* pv. *glycinea* and Subtractive Hybridization-Based Comparative Genomics with Nine Pseudomonads

**DOI:** 10.1371/journal.pone.0016451

**Published:** 2011-01-27

**Authors:** Mingsheng Qi, Dongping Wang, Carl A. Bradley, Youfu Zhao

**Affiliations:** Department of Crop Sciences, University of Illinois at Urbana-Champaign, Urbana, Illinois, United States of America; National Institutes of Health, United States of America

## Abstract

Bacterial blight, caused by *Pseudomonas savastanoi* pv. *glycinea* (*Psg*), is a common disease of soybean. In an effort to compare a current field isolate with one isolated in the early 1960s, the genomes of two *Psg* strains, race 4 and B076, were sequenced using 454 pyrosequencing. The genomes of both *Psg* strains share more than 4,900 highly conserved genes, indicating very low genetic diversity between *Psg* genomes. Though conserved, genome rearrangements and recombination events occur commonly within the two *Psg* genomes. When compared to each other, 437 and 163 specific genes were identified in B076 and race 4, respectively. Most specific genes are plasmid-borne, indicating that acquisition and maintenance of plasmids may represent a major mechanism to change the genetic composition of the genome and even acquire new virulence factors. Type three secretion gene clusters of *Psg* strains are near identical with that of *P. savastanoi pv. phaseolicola* (*Pph*) strain 1448A and they shared 20 common effector genes. Furthermore, the coronatine biosynthetic cluster is present on a large plasmid in strain B076, but not in race 4. *In silico* subtractive hybridization-based comparative genomic analyses with nine sequenced phytopathogenic pseudomonads identified dozens of specific islands (SIs), and revealed that the genomes of *Psg* strains are more similar to those belonging to the same genomospecies such as *Pph* 1448A than to other phytopathogenic pseudomonads. The number of highly conserved genes (core genome) among them decreased dramatically when more genomes were included in the subtraction, suggesting the diversification of pseudomonads, and further indicating the genome heterogeneity among pseudomonads. However, the number of specific genes did not change significantly, suggesting these genes are indeed specific in *Psg* genomes. These results reinforce the idea of a species complex of *P. syringae* and support the reclassification of *P. syringae* into different species.

## Introduction

*Pseudomonas syringae* is a fluorescent pseudomonad clustering within rRNA-similarity group I of the genus *Pseudomonas* [1]. The species *P. syringae* comprises a group of plant-associated bacteria that either act as epiphytes or as plant pathogens causing important diseases with significant economic consequences [2], [3], [4]. Although the *P. syringae* species as a whole causes plant diseases on a multitude of agriculturally important plant species, individual *P. syringae* strains typically exhibit a very high degree of host specificity, infecting only a few plant species or even a few genotypes of a single species [5], [6]. The *P. syringae* species is thus subdivided into more than 50 pathovars, which are mostly described on the basis of plant host range [4]. A comprehensive genetic analysis has indicated the existence of nine discrete genomospecies on the basis of total DNA-DNA homology and ribotyping within the species of *P. syringae* [5]. Among them, genomospecies 2 is re-classified as *P. savastanoi*, including pvs. *savastanoi*, *glycinea*, *tabaci*, and *phaseolicola* [4], [5]. Thus, the *P. syringae* species is genetically diverse, presumably due to adaptation of individual pathovars to their respective host plant environment [7], [8], [9], [10], [11], [12], [13], [14].

In the past decade or so, significant progress has been made in unveiling the mechanisms of pathogenesis of *P. syringae* and other plant pathogens [15], [16], [17]. With either complete or draft genome sequences of *P. syringae* pathovars currently available, this organism is an attractive model for molecular studies of plant-pathogen interactions [18], [19], [20]. A functional hypersensitive response and pathogenicity (*hrp*) type III secretion system (T3SS) that directs the delivery of effector proteins into host cells has been shown to be the key pathogenicity factor required for *P. syringae* to colonize and parasitize host plants [21], [22], [23], [24], [25]. While the complete repertoire of effectors of any one *P. syringae* strain is still unknown, several recent studies have revealed that this number can be as large as 58 in *P. syringae* pv. *tomato* DC3000 [21], [23], [24], [26], [27], [28]. To date, more than 150 effector genes have been identified in *P. syringae* (http://pseudomonas-syringae.org/), and it has been suggested that variations in host specificity may be due to differences in the effector complement of individual *P. syringae* strains [21], [23], [24], [27], [28].

Soybean (*Glycine max*), one of the world's largest providers of protein and oil, is a major crop in the United States, which accounts for about 40% of the soybeans produced in the world. Worldwide, consumption of soybean in human diets utilizes nearly 1% of the crop. This percentage continues to increase as improvements in tastefulness of foods with soybean as a major component occur and as soybean has been recognized as a health food. In addition, approximately 60% of soybeans are used in animal feed. Biodiesel, a fuel used by many city buses and other large vehicles, is usually made from soybeans in the U.S. The demands for soybean production are expected to continue to increase as the world population increases. However, diseases are continuing to be a limiting factor in soybean production. Bacterial blight, caused by *Pseudomonas savastanoi* pv. *glycinea* (*Psg*), is a common bacterial disease of soybean and occurs in most soybean grown areas [29], [30]. Yield losses due to bacterial blight disease of soybean estimate at 4 to 40% in the U.S [29], [30], [31], [32].

Many plant pathogenic bacteria, such as *Psg*, are subdivided into races based on their reactions to some or a set of host cultivars. The first race delimitation in *Psg* was made by Cross and colleagues in 1966 [31], who identified seven races based on their reactions on seven differential soybean cultivars [32], [33], [34]. It has been more than a quarter of century since the first avirulence gene (*avrA1*) was cloned from race 6 of *Psg* by Dr. Staskawicz and colleagues in 1984 [35]. In the subsequent years, two more avirulence genes (*avrB1* and *avrB2*, formerly *avrB and avrC*) were characterized from races 0 and 1 in 1987 and 1988, respectively [36], [37], [38], [39]. The existence of differential cultivars of soybean and distinct races of *Psg* is an indication of a gene-for-gene interaction [40], [41]. Indeed, several R genes in soybean have been identified that recognize the corresponding *avr* gene product in *Psg*. Genetic studies have demonstrated that *Rpg2*, *Rpg1-b* (*Rpg1*), *Rpg3*, and *Rpg4* in soybean specifically interact with *avrA1*, *avrB1*, *avrB2* and *avrD1* genes of *Psg*, respectively [40], [41], [42], [43], [44], [45]. However, recent studies have shown that field isolates of *Psg* in Illinois and elsewhere are either predominantly race 4 or closely related to race 4 [32], [33], [34], which infect all cultivars currently available, indicating field selection or host resistance has been a major factor in the survival of *Psg* races in soybean fields. On the other hand, breeding for disease resistance in plants has been a major means of disease control. However, resistant sources have not been identified for *Psg* race 4. Research has since been lagged behind mainly due to lack of genome sequence of the pathogen.

Complete genome sequence for three pathovars of phytopathogenic pseudomonads on tomato and bean are available include *P. syringae* pv. *tomato* (*Pto*) DC3000, *P. savastanoi* pv. *phaseolicola* (*Pph*) 1448A and *P. syringae* pv. *syringae* (*Psy*) B728a [8], [9], [10]. Draft genome sequences are also available for at least 6 other pathovars: *P. syringae* pv. *aesculi* (*Pae*) 2250 on horse chestnut and Indian chestnut, *P. savastanoi* pv. *savastanoi* (*Psv*) NCPPB 3335 on olive, *Psy* FF5 on ornamental pear, *Pto* T1 on tomato, *P. syringae* pv. *tabaci* (*Pta*) ATCC 11528 on wild tobacco and *P. syringae* pv. *oryzae* (*Por*) 1-6 on rice [7], [11], [12], [13], [14]. Based on the phylogenetic study, these seven pathovars belong to four distinct genomospecies and groups [5], [6]. Among them, two strains of *Psy* belong to genomospecies 1 and group 2. Two strains of *Pto* belong to genomospecies 3 and group 1, and *Por* belongs to genomospecies 4 and group 4. *Pph* 1448A, *Pae* 2250, *Pta* ATCC 11528 and *Psv* NCPPB 3335 belongs to the genomospecies 2 and group 3, which also includes *Psg*.

The goals of this study were: 1) to obtain genome sequences of a field isolate from Illinois and a race 4 strain isolated in the 1960s; 2) to conduct *in silico* subtractive hybridization-based comparative genomic analyses to compare the genomes of two *Psg* strains as well as with nine other sequenced phytopathogenic pseudomonads. We have generated deep coverage, good quality draft genome sequences of *Psg* strains, B076 and race 4, using 454 pyrosequencing. The draft genome sequences were annotated and analyzed for the presence of genomic regions unique to previously sequenced *P. syringae* strains. Our data provide a foundation for detailed functional analyses of host specificity and virulence mechanisms among the *Psg* strains and other *P. syringae* pathovars.

## Results and Discussion

### Bacterial strain selection for sequencing

We have previously screened more than 90 Psg strains isolated from soybean fields in Illinois on nine differential soybean varieties [34]. The majorities of these field isolates showed similar reactions as reported for race 4 of Psg, i. e. causing disease. We also checked whether these field isolates contained known avirulence genes such as avrA1, avrB1, avrB2 and avrD1 using PCR. Our results showed that, avrA1, avrB1 and avrB2 did not exist in all field isolates, with the exception of avrD1, which was present in all isolates (data not shown). These results suggest that races 0, 1, and 6 may not be present in the fields in Illinois, indicating host selection may play a role in the survival of Psg races. In order to identify genetic traits that contribute to virulence, we initiated an effort to sequence a representative strain of recent field isolates. We also decided to sequence the race 4 strain, which was isolated in the 1960s [31], with the objective of comparing a current field isolate with the one from the early 1960s. This comparison may shed light on the genetic diversity of the pathogen.

### Draft genome sequencing data for race 4 and B076

The draft genome sequences for *Psg* race 4 and B076 strains were obtained using 454 pyrosequencing with both shotgun and pair-end libraries. The *Psg* race 4 genome assembly yielded 693 contigs (>100 bp), of which 448 large contigs (>500 bp) comprised 28 scaffolds (maximum scaffold length  = 1,164 kb). The *Psg* B076 genome assembly yielded 1481 contigs (>100 bp), of which 802 large contigs (>500 bp) comprised 17 scaffolds (maximum scaffold length  = 5,312 kb). After manual gap-closing by PCR and re-sequencing using Sanger sequencing, the *Psg* B076 and race 4 draft genomes yielded 104 (56 belong to chromosome sequences) and 108 (79 belong to chromosome sequences) supercontigs, respectively. The total length was 6,236,653 and 5,905,211 bp for B076 and race 4, with a 57.81% and 57.93% G+C content, respectively. The characteristics of the two draft genomes are listed in Table 1. Based on the calculated sequence coverage and comparison with other *Pseudomonas* genomes (see below), we believe that the vast majority of genes are present in the current draft, though the draft genome sequence of race 4 is not as complete as that of B076. The sequences of the supercontigs have been deposited in GenBank under the accession numbers AEGH00000000 and AEGG00000000 for race 4 and B076, respectively. The version described in this paper is the first version, AEGH01000000 (race 4) and AEGG01000000 (B076).

**Table 1:** General features for the *Pseudomonas savastanoi* pv. *glycinea* draft genomes.

*Psg* strain	B076	Race 4
No. of sequences aligned	3,159,883	689,085
No. of bases in aligned sequences	905,980,054	134,123,449
No. of large contigs (>500 bp)	802	448
Largest contig size (bp)	215,091	128,993
No. of scaffolds	17	28
Largest scaffold size (bp)	5,103,126	1,164,000
Total size (bp)	6,236,653	5,905,211
G+C content (%)	57.81%	57.93%
Calculated genome coverage	137	22.8

**Figure 1:**
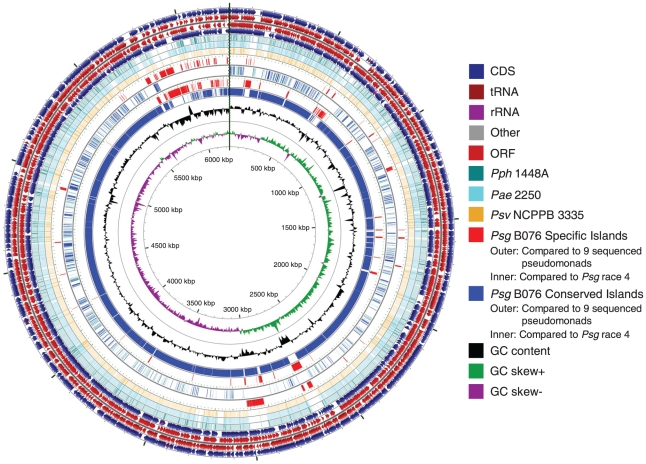
Features of *Pseudomonas savastanoi* pv. *glycinea* strain B076 draft genome. The circles indicate artificial chromosome (concatenated with chromosome and plasmid contigs). The genes, ORFs and RNAs on both directions, are depicted on the outermost four circles of the map, respectively. Only ORFs containing more than 300 codons are shown. The fifth to seventh circles represent the BLASTN comparison of *Psg* B076 against *Pph* 1448A, *Pae 2250*, and *Psv* NCPPB3335 genomes, respectively (BLASTN E value <10^−10^). The eighth to eleventh circles indicate the specific and conserved genomic islands of *Psg* compared to 9 sequenced *P. syringae* strains and *Psg* race 4, respectively. Red: specific islands; Blue: conserved islands. Circular genome map was generated by CGview [86].

**Figure 2:**
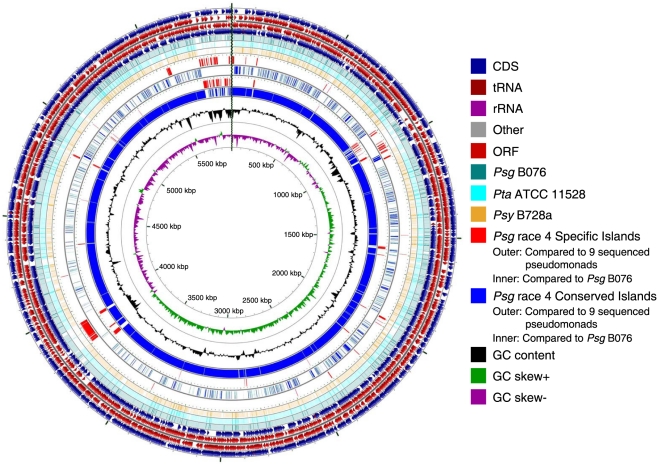
Features of *Pseudomonas savastanoi* pv. *glycinea* race 4 draft genome. The circles indicate artificial chromosome (concatenated with chromosome and plasmid contigs). The genes, ORFs and RNAs on both directions, are depicted on the outermost four circles of the map, respectively. Only ORFs containing more than 300 codons are shown. The fifth to seventh circles represent the BLASTN comparison of *Psg* race 4 against *Psg* B076, *Pta* ATCC 11528, and *Psy* B728a genomes, respectively (BLASTN E value <10^−10^). The eighth to eleventh circles indicate the specific and conserved genomic islands of *Psg* race 4 compared to 9 sequenced *P. syringae* strains and *Psg* B076, respectively. Red: specific islands; Blue: conserved islands. Circular genome map was generated by CGview [86].

Using the NCBI Prokaryotic Genome Automatic Annotation Pipeline (PGAAP), 5,578 potential protein-coding genes were predicted for B706, of which 1526 (27.4%) were annotated as hypothetical proteins or proteins of unknown function (Fig. 1 and Table 2). 5,209 potential protein-coding genes were predicted for race 4, of which 1321 (25.4%) were annotated as hypothetical proteins or proteins of unknown function (Fig. 2 and Table 2). These numbers are comparable with other sequenced pseudomonads reported previously [7], [8], [9], [10], [11], [12], [13], [14].

**Table 2:** Predicted distribution of genes and proteins in Psg draft genomes.

Comparisons	Total genes	BLAST	Conserved genes#	Specific genes#	Other genes #	No. of SI**	SI Size (kb)**
B076 versus Race 4	5578	N	4986	485	107	42	481.7
		P	4934	541	103	51	
Race 4 versus B076	5209	N	4944	190	75	23	217.7
		P	4916	233	60	21	
B076 versus 9 genomes*	5578	N	1250	374	3954	29	405.3
		P	2168	394	3016	31	
Race 4 versus 9 genomes*	5209	N	1251	245	3713	25	297
		P	2166	252	2791	24	

# : Conserved, specific and other genes refer to genes with homology value (H-value) more than 0.81, less than 0.42, and between 0.42 and 0.81, respectively. A gene being conserved indicates that H-value exceeds the threshold in both or all genomes compared.

*: nine genomes refer to *Pph* 1448A, *Pae* 2250, *Psv* NCPPB 3335, *Pta* ATCC 11528, *Psy* B728a, *Psy* FF5, *Pto* DC3000, *Pto* T1 and *Por* 1–6 as described in Figures 6 and 7.

**SI: Specific islands refer to strain-specific genomic regions with more than 3 predicted genes (3 kb). ND: not determined.

### Genome order and conservation

**Figure 3:**
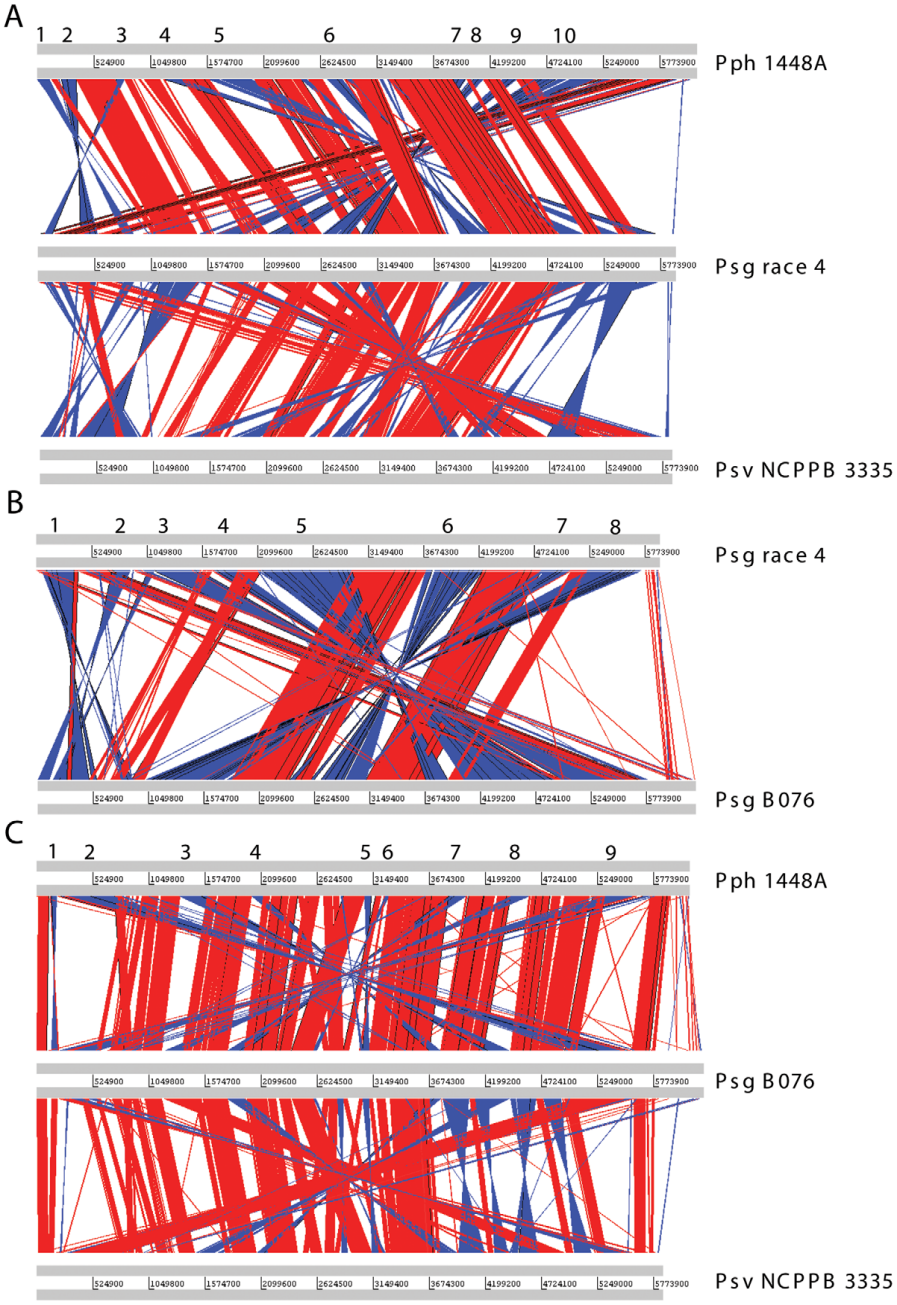
Pairwise alignments between the draft genomes of *Pseudomonas savastanoi* pv. *glycinea* strains and the genomes of *Pph* 1448A and *Psv* NCPPB 3335. BLASTN analyses were performed using WebACT and displayed with the ACT software. The red bars indicate collinear regions of similarity, and the blue bars indicate regions of similarity that are inverted in their relative orientations. Only matches larger than 3 kb are shown. (A) *Psg* race 4 against *Pph* 1448A and *Psv* NCPPB 3335; (B) *Psg* race 4 against B076; (C) *Psg* B076 against *Pph* 1448A and *Psv* NCPPB 3335. Numbers indicate major inversions in the genomes of *Psg* strains with respect to *Pph* 1448A.

It is well known that the genomes of pseudomonads are heterogeneous due to extensive recombination and inversion [46], [47]. To investigate how diverse or conserved among *Psg* and other sequenced *P. syringae* strains, the draft genomes of *Psg* strains were aligned with each other and with the genomes of two close related strains, *Pph* 1448A [10] and *Psv* NCPPB 3335 [14]. As expected from previous comparisons of *P. syringae* genomes [10], [14], these two genomes shared extensive regions of synteny (gene order) as well as sequence conservation with *Psg* genomes, except for nine and ten major inversions in *Psg* B076 and race 4 as compared to *Pph* 1448A, respectively (Fig. 3A and 3C). By comparing the two strains of *Psg*, eight major inversions were observed (Fig. 3B). This result suggested that, although most genome sequences and gene contents are conserved among pseudomonads, many inversion incidents occurred in *Psg* genomes, which may represent the genetic diversification within the *Psg* population.

### *Psg* is phylogenetically closer to *Pph* than to *Psv*, *Pae*, or *Pta*

**Figure 4:**
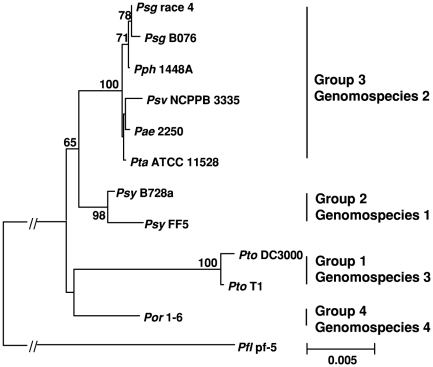
Evolutionary relationship of Psg strains to other nine sequenced Pseudomonas syringae strains. Phylogenetic tree was reconstructed from concatenated sequences of six housekeeping proteins (3154 aa) using Neighbor-Joining (NJ) method. The four major groups as described previously [6] are labeled next to the relevant clades. Genomospecies is defined as previously described by Gardan et al. 1999 [5]. Bootstrap scores greater than 60 are given at each node. The scale bar represents 0.005 amino acids substitutions per site. *Pseudomonas fluorescens* strain pf-5 was used as an outgroup.

*Psg* belongs to group 3, genomospecies 2 as defined by early evolutionary studies based on the concatenation of housekeeping genes and DNA-DNA hybridization [5], [6]. We reconstructed a phylogenetic tree based on the concatenation of six housekeeping genes for nine pathovars of sequenced pseudomonads (Fig. 4). Our phylogenetic analyses confirmed previous defined groups and its corresponding genomospecies [5], [6]. Within the group 3, *Psg* is phylogenetically closer to *Pph* 1448A, a common bean (*Phaseolus vulgaris*) pathogen, than to other pathovars, such as *Psv*, *Pae*, and *Pta*. These results and previous reports support the idea that strains from all woody plants such as *Pae* and *Psv* form separate clade within the group [13]. These results also suggest that pseudomonads that infect similar plant hosts, such as legume and woody plants, may have some evolutionary connection or may be descendents of a monophyletic group.

### Comparative analyses of two *Psg* genomes

As shown in Fig. 4, *Psg* strains B076 and race 4 were clustered in a clade with *Pph* 1448a, but these two *Psg* strains were not identical, one isolated about 50 years ago and the other recently isolated from an Illinois field. An *in-silico* subtractive hybridization based-comparative genomic analysis was undertaken using the genome-wide sequence data of these two *Psg* strains, which allowed us to comprehensively identify the genetic variation between them. BLASTN analysis identified 4,986 and 4,944 conserved genes, and 485 and 190 strain-specific genes in *Psg* B076 and race 4, respectively (Table 2). Those specific genes for B076 and race 4 were distributed in 42 and 23 specific islands (SIs), each of which contains more than three specific genes, with the size of 481.7 and 217.7 kb, respectively (Table 2). Meanwhile, BLASTP analysis identified 4,934 and 4,916 conserved proteins, and 541 and 233 strain-specific proteins in *Psg* B076 and race 4, respectively (Table 2). When comparing the list of specific genes/proteins, 437 and 163 genes/proteins were found common using BLASTN and BLASTP for strain B076 and race 4, respectively (Table 3, Files S1 and S2). These results indicate that more than 4,900 genes/proteins are highly conserved between these two *Psg* strains, representing about 89% to 94% of the total genes/proteins in two *Psg* genomes. These results also suggest that the genome of *Psg* strains is relatively conserved, though they were isolated, one most recently and the other about 50 years ago. However, genetic variation, especially inversion and recombination, occurs very often in these *Psg* strains, while still maintaining the core genome of the pathogen.

**Table 3:** Protein categories encoded by Psg specific genes identified by both BLASTN and BLASTP.

Comparisons	B076		Race 4	
Protein category	versus 9**	versus Race 4	versus 9**	versus B076
Hypothetical proteins, Putative functional protein	226	263	132	111
Integrase, Helicase, Recombinase Transposases, Phage related, DNA restriction and modification	23	30	16	10
Other conserved functional proteins	45	134*	27	42
Total numbers	294	437	175	163

*: Include the coronatine biosynthetic operons.

**: 9 genomes as described in Table 2.

The major difference between B076 and race 4 is that biosynthesis operons of the phytotoxin coronatine (COR), including coronafacic acid (CFA) and coronamic acid (CMA) biosynthesis genes, and regulatory genes of COR production (PsgB076_27705 to 27810, 27845), are present in B076, but not in race 4. Unlike in *Pto* DC3000, coronatine biosynthetic genes are known to be plasmid-borne in *Psg* [48], it is possible that the plasmid containing the COR cluster is lost in race 4. Coronatine, a non host-specific phytotoxin, has been comprehensively studied in *Psg* and *Pto* DC3000 [48]. Coronatine contributes to virulence and suppresses host innate immunity through promoting jasmonic acid (JA) pathway and suppressing salicylic acid (SA) pathway [18]. Coronatine also promotes the opening of stomata, which is one of the major routes for bacterial pathogens to enter host plants [49], [50].

Genes related to a dicarboxylate transport (Dct) system in B076 are also absent in race 4 (File S1). Both B076 and race 4 possess a dctPQM operon (PsgB076_04431 to 04441; PsgRace4_08995 to 09005), which is conserved with that in *Pph* 1448A. Meanwhile, both B076 and Pph1448A contain a conserved Dct transport system (PsgB076_13422 to13432), but race 4 does not. In aerobic bacteria, dicarboxylate transport A carriers catalyze uptake of C4-dicarboxylates. Dicarboxylate may act as the carbon source of *Pto* when proliferating in the apoplast of plant cells [51]. It has also been reported that C4-dicarboxylate transport mutants of *Rhizobium trifolii* form ineffective nodules on *Trifolium repens* [52]. The mutant strains failed to grow on or transport succinate, fumarate, or malate, indicating this system may supply the intracellular bacteria with dicarboxylates as carbon sources. In addition, strain B076, but not race 4, contains an extra copy of PdxA and DapA (PsgB076_08535; PsgB076_08550), key enzymes for vitamin B6 and lysine biosynthesis, respectively. On the contrary, extra copies of enzymes involved in amino acids, ribose and fatty acids metabolism are found in race 4, but not present in B076 (PsgRace4_17349 to 17354; PsgRace4_17414, PsgRace4_17419, PsgRace4_17434 to 17444; PsgRace4_27705, File S2). These differences of genes involving metabolism indicate that *Psg* strains may have developed or acquired different ways to survive within the host plants, which need to be further verified.

Around half of the strain-specific genes of B076 or race 4 encode hypothetical and putative proteins (Table 3). About 6.9% and 6.1% of B076 and race 4-specific genes encode proteins involved in horizontal gene transfer (HGT), including integrases, helicases, recombinases, transposases, DNA restriction and modification system, phage related proteins and conjugal transfer systems (type IV secretion, see below), suggesting that these differences may be due to the numbers of plasmids present in these strains (See Figs. 1 and 2). These results also indicate that so many inversion events between these two *Psg* strains may be the result of these mobile elements. Detailed lists of specific genes for *Psg* strains could be found in Files S1 and S2.

### Determination of plasmids in B076 and race 4 strains

**Figure 5:**
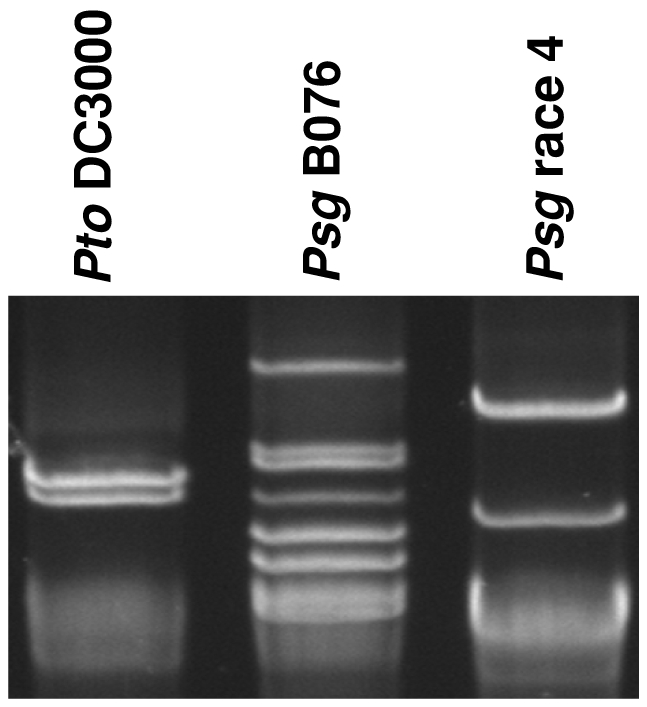
Large plasmid profiles of *Pseudomonas savastanoi* pv. *glycinea* strains. Large plasmid profiles were shown using agarose gel electrophoresis to compare the number and relative sizes of native large plasmids present in the two *Psg* strains. Plasmids of *Pto* DC3000 (pDC3000A and pDC3000B: 73.7 and 67.5 kb, respectively) were included as control.

In order to clarify whether those specific genes are the content of extra plasmids, the number of native plasmids in both B076 and race 4 strains were determined (Fig. 5). The race 4 strain contains five plasmids with unclosed size of *ca* 100, 62, 11, 8, and 4 kb, respectively (contigs 018 to 035; 098 to 108); and the B076 strain contains seven plasmids with unclosed size of *ca* 111, 87, 80, 67, 53, 35, and 8 kb, respectively (from contigs 057 to 104). Only three small plasmids (4 to 8 kb) were totally closed. These results indicate that B076 may have acquired several large plasmids, such as the one containing the coronatine biosynthetic operon. Interestingly, about 49% of specific genes identified in B076, while about 29% of those specific genes identified in race 4 come from plasmids (Figs. 1 and 2 and Files S1 and S2). These results suggest that acquisition and maintenance of plasmids may represent a major mechanism for pseudomonads to change their genetic composition of the genome and even acquire new virulence factors [53]. Interestingly, the B076 strain contains the highest number of plasmids in all phytopathogenic pseudomonads sequenced so far.

Initial genomic analyses of plasmids from pseudomonads have provided glimpses of the gene content, and similarities and differences among different plasmids from varied *P. syringae* pathovars [54], [55], [56], [57], [58], [59]. These studies indicate that genes encoding effector proteins of importance to host-pathogen interactions, other determinants involved in virulence and epiphytic fitness of *P. syringae*, and determinants increasing strain survival on plants sprayed with agricultural bactericides have been identified on plasmids from various pathovars of *P. syringae* [53]. Recent genome sequence analyses also reveal that many effectors are on various plasmids [8], [10], [13], [14]. A recent study on the evolution of type III effectors in *P. syringae* also provided evidence for the recent horizontal acquisition of 11 of 24 identified effector gene families [60]. On the other hand, the biosynthetic gene cluster for the phytotoxin coronatine from *P. syringae* pv. *glycinea* strain 4180 is also on plasmid pPG4180A [48]. Our analyses of plasmids indicate that, not only the coronatine gene cluster, but also *Psg* specific-effector *hopZ1b* and other effector genes such as *hopAV1*, *hopD1*, *hopQ1*, *avrRps4*, *hopAB1*, and *avrPto1* are plasmid-borne (see below). Many of these effectors are surrounded by insertion sequences (transposases), indicating that horizontal transfer has likely played a major role in shaping the repertoire of type III effectors within individual *P. syringae* strains.

### Mobile genetic elements

Both *Psg* genomes contain sequences with high similarity to genes encoding insertion sequences (transposases) found in other sequenced *P. syringae* strains, including IS*801*, IS*Psy1*, IS*Psy2*, IS*Psy3*, IS*Psy4*, IS*Psy5*, IS*Psy6*, IS*Psy12*, IS*Psy14*, IS*Psy16*, IS*Psy18*, IS*Psy19*, IS*Psy20*, IS*Psy21*, IS*Psy24*, IS*Psy25*, and IS*Psy26*. In addition, both *Psg* genomes contain at least 43 genes encoding integrases and resolvases, suggesting a high rate of recombination or horizontal exchange of genetic materials. However, it is not possible for us to accurately estimate the copy number of different insertion sequences, due to incompleteness of the genome sequences. Insertion sequences (IS) are one of the main reasons why many *Pseudomonas* sequence projects are unable to close all the gaps, including in our case. The abundance of IS sequences may also explain in part why rearrangements in the genomes of pseudomonads occur very often [61].

### The T3SS apparatus and its effectors

It is well documented that T3SS plays a critical role in the virulence of many gram-negative bacteria including *P. syringae* [16]. In the sequenced pseudomonad genomes, *Pph* 1448A [10], *Pae* [13], *Psv* [14], *Pta* [11] and *Por* [12], but not *Pto* or *Psy*, contain two distinct T3SSs: the complete Hrp T3SS pathogenicity island (PAI), which is responsible for delivering effectors into plant cells, and a second incomplete system, whose function is still unknown [10], [13]. Both T3SS gene clusters are highly conserved in *Psg* strains, and they are almost identical to those found in *Pph* 1448A (Figure S1). Our phylogenetic analyses showed that the incomplete T3SS is clustered together with those found in *Rhizobium* (data not shown), suggesting that they may belong to a distinct T3SS group [16].

It has been suggested that variations in host specificity may be due to differences in the effector complement of individual *P. syringae* strains [21], [23], [24], [27], [28]. The effector repertoires of *Psg* strains were identified and compared to those previously reported in *P. syringae* strains [21], [23], [24], [27], [28]. Table 4 lists the effector genes of *Psg* strains shared in other sequenced plant-pathogenic pseudomonads. We also separated effectors into groups based on the evolutionary relationship of sequenced phytopathogenic pseudomonads (Fig. 4). Group 1 includes 6 *hop* genes that are conserved in pathovars of genomospecies 2. Among them, two effector genes, *avrE1* and *hopI1*, are also conserved in genomospecies 1 and 3 strains, including *Psy* and *Pto*; whereas the other four exist in genomospecies 4 (*Por*). The five effector genes of group 2 are conserved in *Psg*, *Pph* 1448A, *Pae* 2250 and *Psv* NCPPB 3335. Group 3 consists of nine effector genes, which are shared by *Psg* and *Pph* 1448A strains, but sporadically found in other pathovars. It is tempting to speculate that this group of effectors might have a specific role during interaction with legume host plants.

**Table 4:** Effector genes in *Psg* strains compared to other sequenced *P. syringae* strains.

G*	Effector genes	*hrp* box	Genomo-species 2				Genomo-species 1		Genomo-species 3		Genomo-species 4
			*Pph* 1448A	*Pae* 2250	*Psv*NCPPB 3335	*Pta* 11528	*Psy* B728a	*Psy* FF5	*Pto* DC3000	*Pto* T1	*Por* 1-6
1	*avrE1*	+	+	+	+	+	+	+	+	+	-
	*hopI1*	+	+	+	+	+	+	+	+	+	-
	*hopR1*	+	+	+	+	+	-	-	+	+	+
	*hopV1*	+	+	+	+	+	-	-	+	-	+
	*hopAE1*	+	+	+	+	+	+	-	-	+	+
	*hopAS1*	+	+	+	+	+	-	-	-	+	+
2	*hopD1*	+	+	+	+	-	-	-	+	+	-
	*hopG1*	+	+	+	+	-	-	-	+	-	-
	*hopQ1*	+	+	+	+	-	-	-	+	-	-
	*hopAB1*	-	+	+	+	-	-	-	-	-	-
	*hopAF1* #	+	+	+	+	-	+	-	+	+	-
3	*avrB4-1*	+	+	+	-	-	-	-	-	-	-
	*avrD1*	+	+	-	-	-	-	-	-	+	-
	*hopX1*	+	+	+	-	+	+	-	+	-	-
	*hopW1-1* &	+	+	-	-	-	-	-	-	-	-
	*avrRps4* &	- IS	+	-	-	-	-	-	-	-	-
	*hopAT1*	-	+	-	-	-	-	-	-	-	-
	*hopAV1*	+	+	-	-	-	-	-	-	-	-
	*hopAW1* #	+	+	-	-	-	-	-	-	-	-
	*hopAU1*	+	+	-	+	-	-	-	-	-	-
4	*hopC1*	+	-	-	-	-	-	-	+	+	-
	*hopH1*	+	-	-	-	-	+	-	+	+	-
	*hopZ1b*	+	-	-	-	-	-	-	-	-	-
	*avrPto1*	+	-	+	-	-	+	-	+	-	-

*Group 1: effector genes common in genomospecies 2; Group 2: effector genes shared by *Psg*, *Pph* 1448A and *Pta* ATCC 11528. Group 3: effector genes shared by *Psg* and *Pph* 1448A; Group 4: *Psg* specific effector genes, but not in other genomospecies 2 strains except for *avrPto1.*

- IS: IS*Psy* insertion in the promoter region.

&: effector genes found in race 4 but not in B076, dash underlined.

#: effector genes found in B076 but not in race 4, solid underlined.

Group 4 contains four effector genes, *hopC1*, *hopH1*, *avrPto1* and *hopZ1b*, which appear to be *Psg* strain specific among the 5 sequenced strains of genomospecies 2, with exception of *avrPto1* in *Pae*; while *hopZ1b* is *Psg* specific among all 9 sequenced *P. syringae* strains. It has been reported that the *hopZ* effector family is widely distributed in *P. syringae* strains and is ancient to *P. syringae* [62], [63]. It has also been reported that *hopZ1b* is present in *P. sesame*, another member of the genomospecies 2; whereas *hopZ1a*, *hopZ1c*, *hopZ2*, and *hopZ3* are present in various pseudomonad strains including *Psy* B728a [62]. Indeed, recent studies have proven that HopZ1 are differentially recognized by plant resistant systems in different host plants [63], [64], [65].

It is worth noting that 20, 14, 12, and 7 effector genes of *Psg* can be found in *Pph* 1448A, *Pae* 2250, *Psv* NCPPB3335 and *Pta* 11528, respectively (Table 4). When comparing the two *Psg* strains, *hopAW1* and *hopAF1* are only present in *Psg* B076, while *avrRps4* and *hopW1-1* can only be found in *Psg* race 4. It is possible that these effectors may play specific roles in the interaction of *Psg* strains with different soybean cultivars. Future studies are needed to determine whether they play a role in host specificity.

Moreover, using effector prediction program EffectiveT3 (http://www.effectors.org/), we were able to predict 13 and 12 candidate genes in the genomes of *Psg* B076 and race 4, respectively, which do not share any sequence similarity with known effectors and have high bit score and secretion signal. Among them, two (PsgB076_07412 and 23576) and one (PsgRace4_06847) candidate gene from *Psg* B076 and race 4 are strain specific compared to the 9 sequenced *P. syringae* strains. However, these candidate genes need to be experimentally confirmed about their identities as Hop proteins. Besides, several truncated or pseudo effector genes exist in the genomes of *Psg*, including *hopM1*', *hopAA1*', *hopPtoU*', and *hopAC1*'. Other genes in *Psg* strains such as *hopJ*, *hopAJ1*, *hopAJ2*, and *hopAN1* are no longer listed as *Pseudomonas hop* genes in the Hop Database (http://pseudomonas-syringae.org/).

### Other protein secretion systems

Besides type III secretion system and its effectors, structural genes involved in the biosynthesis of secretion systems such as type I, type II, type IV, TAT and type VI were identified in the *Psg* genomes. Genes encoding for these secretion systems are listed in File S3. One of the interesting findings is that *Psg* B076 genome contains multiple copies of genes encoding for type IVA Vir secretion systems (VirB1 to VirB11 and VirD1), but only one copy in *Psg* race 4. Both *Psg* genomes have one complete Type IVB Tra system, which is conserved with that in *Pto* DC3000. These differences may also be due to the plasmid contents in these two *Psg* strains. A recent comparative genomic approach examining 31 plasmids revealed that the plasmids could be subdivided into major groups based on the conjugative transfer (type IV secretion) system encoded [53], [57]. Interestingly, in strain B076, several type IVA (VirB-VirD4) systems co-exist.

### Other potential virulence systems

Besides T3SS and non host-specific toxin coronatine, other known virulence determinants in plant pathogenic pseudomonads include cell wall-degrading enzymes, extracellular polysaccharides, iron-uptake systems (siderophores), resistance mechanisms to plant-derived antimicrobials, adhesion and the general processes of motility and chemotaxis.

Six putative cell wall-degrading enzymes, including one cellulase (PagB076_04011, PagRac4_00485), 2 pectate lyase (PsgB076_04196, 10510; PsgRace4_09732, 15814), one pectin lyase (PsgB076_05845; PsgRace4_25621) and 2 polygalacturonase (PsgB076_27232, 28135; PsgRace4_ 23510, 27825) are found in the genomes of *Psg.* Exopolysaccharides, which are components of the bacterial capsule, play an important role in both adhesion and protection of bacterial cells from external stresses [66]. In phytopathogenic pseudomonads, the exopolysaccharide alginate has been reported as an epiphytic fitness and virulence factor in relation to the production of water soaked lesions [67], [68], [69]. A set of 20 genes involved in alginate biosynthesis exist in both *Psg* genomes (File S3). In addition, levansucrase, an enzyme required for biosynthesis of the polysaccharide levan, seems to have a role in the early phase of infection by creating a separating layer between bacteria and plant cell to prevent pathogen recognition by host plant [70]. *Psg* B076 genome contains three levansucrase genes (PsgB076_00457, 10300, 29060), while *Psg* race 4 genome has two (PsgRace4_03819, 15609).

Cell surface associated factors such as pili increase pathogenic bacteria survival on the host surface [71]. 28 genes with annotations related to the biosynthesis of type IV pili, a major group of adhesion factors in pseudomonads [71], are found in both *Psg* strains (File S3). *Psg* B076 contains one putative filamentous hemagglutinin (PsgB076_03035), but none is found in *Psg* race 4. Other potential virulence-related factors are also identified in the *Psg* genomes, including siderophores, quorum sensing and multidrug transporters, which are listed in File S3.

### *in-silico* subtractive hybridization based-comparative genomic analyses with nine sequenced phytopathogenic *Pseudomonas syringae* strains

**Figure 6:**
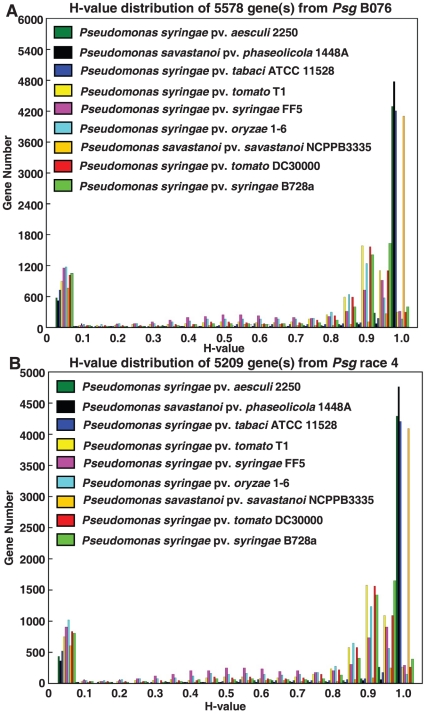
Histogram of BLASTN-based homology value distribution of all predicted CDSs in *Psg* strains compared to nine sequenced *Pseudomonas syringae* genomes. The H value reflects the degree of similarity in terms of length of match and the degree of identity at nucleotide level between the matching CDS in the subject genome and the query genomes with E value <10^−10^. Specific and conserved genes are arbitrarily defined as previously described [72] for those genes with H value less than 0.42 and more than 0.81, respectively. Other genes are defined with H values between 0.42 and 0.81. (A) *Psg* B076 against nine phytopathogenic pseudomonads; (B) *Psg* race 4 against nine pseudomonads, including *P. syringae* pv. *tomato* (*Pto*) DC3000, *Pto* T1, *P. savastanoi* pv. *phaseolicola* (*Pph*) 1448A, *P. syringae* pv. *syringae* (*Psy*) B728a; *Psy* FF5, *P. syringae* pv. *aesculi* (*Pae*) 2250, *P. savastanoi* pv. *savastanoi* (*Psv*) NCPPB 3335, *P. syringae* pv. *tabaci* (*Pta*) ATCC 11528 and *P. syringae* pv. *oryzae* (*Por*) 1–6.

As a newly developed tool for comparative genomic analysis, mGenomeSubtractor is able to run BLAST searches of the reference genome against multiple bacterial genomes (up to 30 user-selected or 10 user-supplied genomes) [72]. Since genome sequences for four genomospecies of pseudomonads are available, we compared both *Psg* genomes to nine previously sequenced phytopathogenic *P. syringae* genomes using both BLASTN and BLASTP. The H-value (Homology-value) distribution of all genes/proteins from *Psg* B076 and race 4 compared to each of the 9 *P. syringae* genomes using BLASTN and BLASTP are shown in Figs. 6 and S2, respectively. The overall trend is very similar between BLASTN and BLASTP results (Figs. 6 and S2) and thus we will only discuss the BLATN data below. Conserved and specific genes/proteins are defined as previously described for those genes/proteins with the H-values of more than 0.81 and less than 0.42, respectively (Figs. 6 and S2) [72]. Interestingly, the majority of conserved genes/proteins in pseudomonads have H-values of more than 0.85, suggesting pseudomonads are evolutionally conserved, and these genes/proteins may represent the core genome among them. On the other hand, the majority of specific genes/proteins in pseudomonads have H-values of less than 0.1, indicating that these specific genes may be acquired recently through horizontal transfer (Figs. 6 and S2).

**Figure 7:**
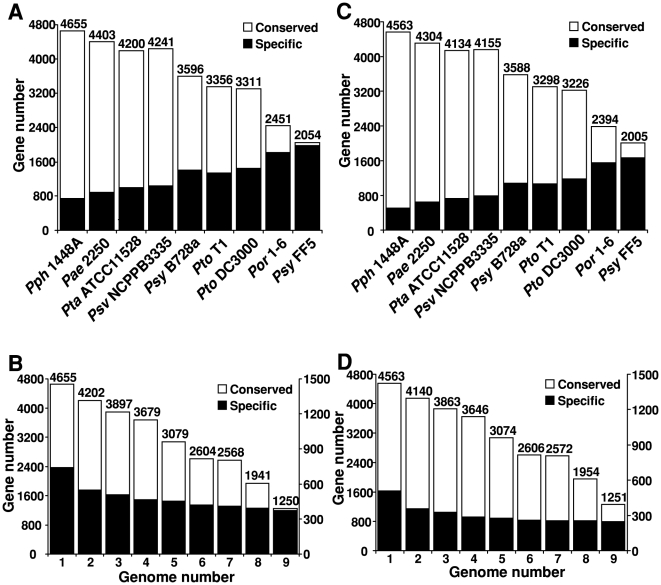
Numbers of conserved and specific genes in Psg genomes compared to nine Pseudomonas syringae genomes individually or by combination of genomes. (A) Numbers of conserved and specific genes in *Psg* B076 compared to each of nine pseudomonad strains as described in Figure 6; (B) Numbers of conserved and specific genes in *Psg* B076 compared to combinations of nine pseudomonads based on results in (A) from left to right., i.e. “1” stands for one genome of *Pph* 1448A, “2” stands for two genomes (*Pph* 1448A and *Pae* 2250), “3” stands for three genomes (*Pph* 1448A, *Pae* 2250 and *Pta* ATCC 11528), and so on; (C) Numbers of conserved and specific genes in *Psg* race 4 compared to each of nine pseudomonad strains as described in Figure 6; (D) Numbers of conserved and specific genes in *Psg* race 4 compared to combinations of nine pseudomonads based on results in (C) from left to right., i.e. “1” stands for one genome of *Pph* 1448A, “2” stands for two genomes (*Pph* 1448A and *Pae* 2250), “3” stands for three genomes (*Pph* 1448A, *Pae* 2250 and *Pta* ATCC 11528), and so on. Numbers on the right Y-axis are for the specific genes in B and D.

The numbers of conserved and specific genes/proteins in *Psg* genomes compared to each of nine genomes of *P. syringae* based on H-value distribution were also determined (Figs. 7AC and S3AC). Based on the number of conserved and specific genes, BLASTN results indicate that *Psg* genomes contain more conserved and less specific genes compared to those members in the same genomospecies than to members of other genomospecies with the exception of *Psy* FF5 (Fig. 7AC), and this trend is basically consistent with their phylogenetic relationship using housekeeping genes (Fig. 4). We reasoned that the exception may be due to the low quality of the *Psy* FF5 draft genome sequence. The major difference between BLASTN and BLASTP results was that the number of conserved proteins with members of other genomospecies dramatically increased, and as a result, the number of specific proteins significantly decreased (Fig. S3AC). These results suggest that nucleotide diversification or different codon usage may occur often among different genomospecies.

Comparisons between *Psg* and other *P. syringae* genomes were also performed by adding one more genome at a time and until all nine genomes, based on their phylogenetic relationship and the number of conserved genes (in order) (Fig.4, Figs. 7AC and S3AC). As shown in Figs. 7BDand S3BD, both the numbers of conserved and specific genes/proteins in *Psg* genomes were steadily decreased when more genomes were added in the comparison. BLASTN results identified only 1,250 and 1,251 conserved genes for *Psg* B076 and race 4, respectively, when subtracted from all nine genome sequences (Table 2, Fig. 7BD). Interestingly, the numbers of conserved genes for the two *Psg* strains are very similar, indicating the vast majority of genes are present in the current draft for both *Psg* genomes. These conserved genes are the “core genome” among the *P. syringae* strains compared. Furthermore, the core genome is more than 3,500 genes when only members of the genomospecies 2 are compared (Fig. 7BD). These results further support the current reclassification of genomospecies 2 into a new species as *P. savastanoi* [5].

Furthermore, the number of specific genes did not change as dramatically as that for the conserved genes when more genomes were included in the subtraction, suggesting that these specific genes are indeed strain-specific for *Psg* strains. 374 and 245 specific genes for *Psg* B076 and race 4, respectively, were found when subtracted from all nine genome sequences using BLASTN (Table 2, Fig. 7BD). When comparing the list of specific genes/proteins, 294 and 175 genes/proteins were found common using BLASTN and BLASTP for strain B076 and race 4, respectively (Files S4 and S5). Among them, 137 (46.6%) and 31 (17.7%) genes/proteins for strain B076 and race 4, respectively, were plasmid-borne, indicating again that plasmids are the main source for pseudomonads to acquire new genetic materials as suggested previously [53]. These genes may represent the true specific genes in these two *Psg* strains compared to the nine sequenced genomes. On the other hand, these results indicate that genes with H-value between 0.81 and 0.42 may increase dramatically, suggesting genes within this category may undergo more changes during evolution than genes in the “core genome”, and the number of genes in both the “dispensable” and “pan-genome” may increase as well when more pseudomonad genomes are compared [73].

Many specific genes from *Psg* were clustered in the genomes. Specific genes/proteins from B076 and race 4 were distributed in 29/31 and 25/24 specific islands (SIs), respectively (Files S4 and S5; Figs. 1 and 2). Among these *Psg* specific genes, more than three quarters encode hypothetical and putative proteins (Table 3). About 7.8% and 3.4% of them encode proteins related to horizontal gene transfer (HGT), including integrases, helicases, recombinases, transposases, DNA restriction and modification system and phage related proteins. For other conserved functional proteins, most of them are shared in both *Psg* B076 and race 4 (Table 4). One specific gene is NAD(P)H-dependent 2-cyclohexen-1-one reductase (Ncr) (PsgB076_13682, PsgRace4_17933). It has been reported that this gene differentially expresses at low temperature and may be involved in the infection of host plants during periods of cold, humid weather conditions, which are the favorite conditions for *Psg* to cause disease [74]. Bacterial blight of soybean is more severe in cold wet conditions than hot warmer conditions [48], indicating that temperature regulation of gene expression is very important for this pathogen to survive and cause disease. It is also true that production of the phytotoxin coronatine is induced at low temperature (18°C) than at high temperature (28°C) [48]. In addition, ethylene forming enzyme (EFE) (PsgB076_27645, PsgRace4_27870) and HopZ1b (PsgB076_27855, PsgRace4_04656, see above) are also found *Psg*-specific in our comparison. However, EFE has been previously reported in *Psg*, *Pph* PK2, *P. syringae* pvs. *cannabina*, *pisi* and *sesame* [75], [76], [77]. These genes may not be specific when more *P. syringae* strains are analyzed and compared.

### Summary

In summary, we have generated high quality draft genome sequences for two *Psg* strains, and bioinformatics and comparative genomic analyses of draft genome sequences have revealed that more than 4,900 genes are highly conserved between them, indicating relatively low genetic diversity between *Psg* genomes, though these strains were isolated, one most recently and the other about 50 years ago. However, comparative genomic analyses also revealed genetic variation, especially inversion and recombination, occurs very often in these *Psg* strains. Strain-specific genes were also identified and the majority of these genes are plasmid-borne, indicating that acquisition and maintenance of plasmids may represent a major mechanism for pseudomonads to change their genetic composition of the genome and even acquire new virulence factors. The effector repertoires and specific effectors such as HopZ1b of *Psg* strains were identified and compared to other pseudomonads, which may determine the host specificity. Other virulence factors such as coronatine toxin, ethylene, and alginate production contribute to the virulence of this pathogen.

*In-silico* subtractive hybridization-based comparative genomic analyses with nine other sequenced phytopathogenic pseudomonads suggest that, the genome of *Psg* strains is more similar to those belonging to the same genomospecies such as *Pph* 1448A than to other phytopathogenic pseudomonads. As more genomes were included in the comparison, the number of highly conserved genes belonging to the core genome decreased dramatically, suggesting diversification of pseudomonads. This diversification also indicates the significant size of genetic information within the *Pseudomonas* pan genome. Furthermore, the number of specific genes did not change significantly when more genomes were subtracted and compared, suggesting these specific genes are indeed unique to *Psg* strains. These results reinforce the idea of a species complex of *P. syringae* and support the reclassification of *P. syringae* into different species.

This multiple genome comparison, the first such comparison in phytopathogenic bacteria as far as we know, may provide a resource for future investigation into the virulence, host specificity and classification of pseudomonads. This approach may also be the basis to define both the true “core genome” and the ever expanding “pan genome” of pseudomonads when more genome sequences are available.

## Materials and Methods

### Growth of bacterial strains, and genomic and plasmid DNA isolation

*Pseudomonas savastanoi* pv. *glycinea* race 4 was a gift from Dr. Brian Staskawicz, University of California, Berkeley. *P. savastanoi* pv. *glycinea* B076 was isolated in 2007 from a diseased soybean leaflet near Champaign, Illinois, USA, and identity was confirmed by PCR and pathogenicity tests. The bacterial strains were differentiated on nine soybean cultivars as described previously to define the races [31], [34]. Bacterial strains were grown on LB medium at 28°C. Genomic DNA was extracted using a modified method of Kinscherf et al. (1991) [78] as described previously. Plasmid DNA was extracted using a modified large scale alkaline lysis method as described previously [57], [79].

### Library construction and genome sequencing

Sample DNA concentration was measured using Nanodrop ND-1000 Spectrophotometer (NanoDrop Technologies, Wilmington, DE) and visualized by agarose gel electrophoresis. Both shotgun and 8kb pair-end libraries were constructed at the W. M. Keck Biotechnology Center for Comparative and Functional Genomics Facility at the University of Illinois. 454 pyrosequencing was carried out with Roche/454 Genome Sequencer FLX-Titanium technology (Roche, Basel, Switzerland) using 20 µg of DNA. A paired-end (PE) library analysis was applied to determine the orientation and relative position of contigs produced by *de novo* shotgun sequencing.

### Genome assembly and annotation

Assembly was performed using the GS De Novo Assembler software provided by 454 Life Sciences. Gap closing was performed using polymerase chain reaction (PCR) and Sanger sequencing. NCBI Prokaryotic Genomes Automatic Annotation Pipeline (PGAAP) (http://www.ncbi.nlm.nih.gov/genomes/static/Pipeline.html) was used to automatically annotate the draft genomes of *Psg*. In brief, gene predictions were done using a combination of GeneMark and Glimmer [80], [81], [82]. Ribosomal RNAs were predicted by sequence similarity searching using BLAST against an RNA sequence database and/or using Infernal and Rfam models. Transfer RNAs were predicted using tRNAscan-SE [83]. In order to detect complete gene sets, a complete six-frame translation of the nucleotide sequence was done and predicted proteins (generated above) were masked. All predictions were then searched using BLAST against all proteins from complete microbial genomes [84]. Annotation was based on comparison to protein clusters and on the BLAST results. Conserved Domain Database and Cluster of Ortholog Group information was then added to the annotation.

### *In silico* subtractive hybridization-based comparative genomics analysis

Genome comparison among genomes of *P. savastanoi* pv. *glycinea* and other sequenced *P. syringae* strains was carried out using mGenomeSubstractor web server [72]. Genomes were also aligned using a genome-wide Blast comparison and visualized through ACT [85]. Graphical views of genome alignments were generated using CGView [86]. Protein sequences of known effectors from Hop Database (http://pseudomonas-syringae.org/) were used for effectors prediction [87]. Effector prediction was also performed using the EffectiveT3 program (http://www.effectors.org/) [88].

### Phylogenetic analysis

To investigate the phylogenetic position of *Psg* within the evolutionary radiation of nine sequenced *P. syringae* pathovars, we used the partial sequences of six housekeeping proteins (AcnB, GapA, GltA, GyrB, Pgi and RpoD) as described previously [6]. The concatenated sequences yielded an alignment with 3,154 sites that could be compared among all strains. Neighbor-joining (NJ) trees were generated in MEGA, version 4.0 [89], using the Poisson correction with 1,000 bootstrap replicates for all sequences.

## Supporting Information

File S1 **Specific gene list of** ***Psg*** **B076 compared to** ***Psg*** **Race 4.**
(XLS)

File S2 **Specific gene list of** ***Psg*** **Race 4 compared to** ***Psg*** **B076.**
(XLS)

File S3 **Putative virulence-related genes in** ***Psg*** **draft genomes.**
(XLS)

File S4 **Specific gene list of** ***Psg*** **B076 compared to 9 sequenced** ***P. syringae*** **strains.**
(XLS)

File S5 **Specific gene list of** ***Psg*** **Race 4 compared to 9 sequenced** ***P. syringae*** **strains.**
(XLS)

Figure S1 **Schematic map of two T3SS pathogenicity islands from four sequenced Pseudomonas syringae strains.** (A) The T3SS clusters of *Psg* B076 and race 4, *Pph* 1448A; *Pto* DC3000 and *Psy* B728a are shown. Same as in *Pph* 1448A, *hopAA1* is a pseudogene and *hopM1* is truncated in *Psg* strains. In the exchangeable effector locus, *hopB1* is shown for *Pto* DC3000; and *avrB3*, *hopX1* and *hopZ3* are shown for *Psy* B728a. (B) An additional incomplete set of T3SS is found in *Psg*, *Pph*, *Pae*, *Psv*, *Pta* and *Por*. T3SCP: Type Three Secretion Component, putative. Phylogenetic analysis indicate that the incomplete T3SCP was closely related to that reported from *Rhizobium* (data not shown), suggesting the potential origin of this T3SCP.
(EPS)

Figure S2 **Histogram of BLASTP-based homology value distribution of all predicted proteins in** ***Psg*** **strains compared to nine sequenced** ***Pseudomonas syringae*** **genomes.** The H-value reflects the degree of similarity in terms of length of match and the degree of identity at amino acid level between the matching protein in the subject genome and the query genomes with E value <10^−10^. Specific and conserved proteins are arbitrarily defined as previously described [72] for those genes with H value less than 0.42 and more than 0.81, respectively. Other proteins are defined with H values between 0.42 and 0.81. (A) Psg B076 against nine pseudomonads; (B) Psg race 4 against nine pseudomonads as described in Figure 6.
(EPS)

Figure S3 **Numbers of conserved and specific proteins in Psg genomes compared to nine Pseudomonas syringae genomes individually or by combination of genomes.** (A) Numbers of conserved and specific proteins in Psg B076 compared to each of nine pseudomonad strains as described in Figure 6; (B) Numbers of conserved and specific proteins in Psg B076 compared to combinations of nine pseudomonads based on results in (A) from left to right., i.e. “1” stands for one genome of Pph 1448A, “2” stands for two genomes (Pph 1448A and Pae 2250), “3” stands for three genomes (Pph 1448A, Pae 2250 and Pta ATCC 11528), and so on; (C) Numbers of conserved and specific proteins in Psg race 4 compared to each of nine pseudomonad strains as described in Figure 6; (D) Numbers of conserved and specific proteins in Psg race 4 compared to combinations of nine pseudomonads based on results in (C) from left to right., i.e. “1” stands for one genome of Pph 1448A, “2” stands for two genomes (Pph 1448A and Pae 2250), “3” stands for three genomes (Pph 1448A, Pae 2250 and Pta ATCC 11528), and so on. Numbers on the right Y-axis are for the specific proteins in B and D.
(EPS)
